# Enhancing *Brassica* microgreen production: Exploring metabolomic variations across growing conditions using targeted and non-targeted analysis

**DOI:** 10.1016/j.fochx.2025.103125

**Published:** 2025-10-10

**Authors:** Arun Kumar, Narpinder Singh, Robin Joshi

**Affiliations:** a Department of Food Science and Technology, Graphic Era (Deemed to be University), Dehradun 248002, India; b Institute for Translational Medicine and Therapeutics, University of Pennsylvania (UPenn), Philadelphia, PA 19104, USA

**Keywords:** *Brassica* microgreens, growing conditions, macroelement, metabolites, sugars

## Abstract

The present study investigated the impact of different photoperiods and temperatures as growing conditions (GCs) on the metabolomic profile of *Brassica* microgreens. The research delves into the intricate chlorophyll content, macroelement composition and metabolomic profiles of *Brassica* species cultivated under varied GC by utilizing targeted and non-targeted compound analysis. Under the G_3_ (a prolonged photoperiod of 22 h with temperatures of 26 °C/20 °C:: light/dark), *Brassica* microgreens demonstrated a rise in chlorophyll content by about 32 %, while microgreen height increased by approximately 20 %. The macroelement composition also varied significantly with GC, notably potassium content increased from 42.93 to 66.18 mg/g under G_3_. The phenolic composition analysis revealed a prominent presence of gallic and ferulic acid in the microgreens, significantly influenced by the GCs. Additionally, sugar profile indicated elevated levels of glucose and sucrose in response to G_3_. The UHPLC-QToF-IMS metabolomic profile highlighted the variation in expression levels of different classes of compounds, specifically (2*R*)-2-Hydroxy-3-butenyl, and 3-Indolylmethyl, which were upregulated under the G_3_.

## Highlights

• Total chlorophyll content increased in response to longer photoperiod.
• The concentration of individual phenolic compounds varied with growing conditions.
• Extended photoperiods elevated *myo*-inositol and glucose levels.
• The expression level of 4-Methoxy-3-indolylmethyl and 3-Indolymethyl increased under longer photoperiod.

## 1 Introduction

Chinese cabbage (*Brassica rapa* L.), a member of *Brassicaceae* family, which is commonly referred to as cruciferous vegetables. These microgreens are nutritionally dense and an excellent source folic acid, vitamin C, vitamin A, Fe, Ca, Cu, Se and Zn (Ağagündüz et al., 2022). These nutrients are fundamental for overall well-being and help reduce the risk of long-term diseases. The major bioactive compounds like glucosinolates, A range of phenolic compounds, comprising flavonoids, anthocyanins and hydroxycinnamic acids, carotenoids, tocopherols, and fatty acids (oleic, stearic, linolenic, linoleic, palmitic and eicosenoic). *Brassica* microgreens are rich sources of health-promoting bioactive compounds, particularly glucosinolates and their hydrolysis products like sulforaphane (Alloggia et al., 2025). Glucosinolates and isothiocyanates in these greens also prevented cancer (Lynn et al., 2006). The anticarcinogenic effects of crucifers were influenced by the amount of glucosinolates they contain, the efficiency of glucosinolate conversion into isothiocyanates, and their bioavailability (Song & Thornalley, 2007). Dereje et al. (2023) reported that glucosinolates exhibited strong antioxidant, anti-inflammatory, and anticancer properties by enhancing detoxification pathways, reducing oxidative stress, and modulating inflammatory responses. Additionally, *Brassica* microgreens also contained high levels of phenolic compounds, vitamins (A, C, E, K), minerals, and chlorophyll, making them nutritionally superior to their mature counterparts. Their bioactive constituents also contributed to cardiovascular health by improving lipid metabolism and endothelial function, and some exhibit antimicrobial and antidiabetic effects (Pawar et al., 2023). In a greenhouse environment with temperatures between 20 and 25 °C and relative humidity of 50–60 %, Chinese cabbage synthesizes metabolites like steroids, terpenes, glucosinolates, and waxes, all of which originate from primary photosynthetic pathways and were affected by environmental factors that also impacted photosynthesis (Baek et al., 2016). Liang et al. (2019) deciphered that the phytochemical content of head cabbages was impacted by agronomic factors and cultivation techniques. The light period played a regulatory factor in the production of glucosinolates and sulphur assimilation, resulting in elevated levels of glucosinolates during daylight hours (Huseby et al., 2013). Zheng et al. (2018) deciphered that the application of supplemental blue light for 10 days prior to harvest led to a noteworthy enhancement in the abundance of phenolic compounds and their antioxidant efficacy. The application of supplemental blue light for 10 days prior to harvest led to a noteworthy enhancement in the levels of phenolic compounds and antioxidant capacity. Brazaitytė et al. (2021) revealed that increased exposure to blue light aided a beneficial impact on the mineral nutrient accumulation in *Brassicaceae* microgreens. These results suggested that utilizing these conditions strategically served as an effective method for enhancing biofortification. According to Gmižić et al. (2023), the temperature at which seedlings grow is a crucial factor in determining their composition and the biological activity of their extracts, and should be considered when growing *Brassica* seedlings.

The studies on impact of growing conditions on the composition of *Brassica* microgreens are very limited. Therefore, this study is the first to explore how different growing conditions influence the composition and metabolomic profile of *Brassica* microgreens, providing insights to improve cultivation practices.

## 2 Materials and methods

### 2.1 Plant material

The *Brassica* microgreens seeds used in the present work were obtained from CSIR-IHBT, Palampur, Himachal Pradesh, India.

### 2.2 *Brassica* cultivation

After a thorough cleansing, *Brassica* microgreen seeds were soaked for 8 h and drained. They were allowed to germinate in darkness for 36 h. Once germination was complete, the seeds were uniformly spread on trays and positioned in a plant growth chamber. *Brassica* microgreen seeds were subjected to four distinct growth conditions with varying photoperiods and temperatures: Control, Growing Condition 1 (G_1_), Growing Condition 2 (G_2_), and Growing Condition 3 (G_3_). The cultivation conditions are described in Table S1 (Supplementary Table). After growing for two weeks, the microgreens were harvested and juiced using a hand-operated screw juicer. The extracted juice was lyophilized, and the resulting lyophilized juice powder (LJP) was used for subsequent analyses.

### 2.3 Total chlorophyll content

The method outlined by Hiscox and Israelstam (1979) was used, with slight modifications by Kumar et al. (2023), to analyze the total chlorophyll content (TCC) of LJPs.

### 2.4 Macroelement composition

The macroelement composition of LJP was analyzed following the protocol outlined by Kumar et al. (2023). Briefly, 0.1 g of LJP was digested with HNO_3_/HCl (1:3) mixture at 150 °C for 2.5 h, then diluted to 50 mL and filtered (0.45 μm). The prepared solution was analyzed using a Metrohm IC system with a conductivity detector and a Metrosep C6 150/4.0 column. Macroelements like sodium, potassium, calcium, and magnesium were separated using a mobile phase of 1.7 mM HNO_3_ and 0.7 mM dipicolinic acid at 0.9 mL/min and 8 MPa, with quantification achieved by comparing retention times to analytical standards and processing data with MagicNet software.

### 2.5 Phenolic profile

The free and bound phenolic compounds were extracted from LJP using a modified method by Kumar, Singh, Kaur, and Joshi (2023). Phenolic compounds (both free and bound forms) were extracted from *Brassica* microgreens' LJPs following a modified method from Kumar, Singh, and Joshi (2023). Free phenolics were obtained by extracting 0.5 g of LJP with 80 % methanol, concentrating the combined supernatants, and redissolving the residue in 1 mL of 80 % methanol. Bound phenolics were subsequently extracted from the leftover residue by alkaline hydrolysis (4 M NaOH for 2 h), acidification, and three successive extractions with an ethyl acetate and diethyl ether mixture (1:1, *v*/v). The organic phase was dried and redissolved in 1 mL of 80 % methanol. Both extracts were stored at 4 °C. Identification was performed using an Agilent 1260 Infinity HPLC system with DAD and a C18 column (250 mm × 4.6 mm). The mobile phase consisted of 0.1 % trifluoroacetic acid in water (A) and 50 % acetonitrile with 0.2 % TFA in water (B). Chromatogram peak areas were integrated using EZChrom Elite 3.2.0. The extracts were dried, redissolved in 80 % methanol, and stored at 4 °C until analysis.

### 2.6 Sugar profile

Using the ion exchange chromatography system (Metrohm 940 Professional IC Vario) with a pulsed amperometric detector and the method described by Kumar, Singh, and Joshi (2023b), the targeted sugars in LJPs were identified. Sugars were separated using a Metrosep Carb 2–250/4.0 column (250 mm × 4.0 mm) with an isocratic eluent of 0.1 mol/L sodium hydroxide and 0.01 mol/L sodium acetate. A flow rate of 0.5 mL/min and a pressure of 15 MPa were maintained. Chromatogram peak areas were quantified using MagIC Net software (version 3.2).

### 2.7 UHPLC-QToF-IMS based non-targeted analysis

The analysis of secondary metabolites in LJP was conducted using a high-resolution Agilent 6560 Ion Mobility System, following the methodology outlined by Kumar et al. (2024). Metabolite elution was carried out using a C18 column (150 mm × 2.1 mm) operated under positive ionization mode. Data acquisition from the LJP was comprehensively analyzed using Mass Profiler Professional software (version B.12.0). Metabolites exhibiting an absolute abundance greater than 4000 counts were selected based on matching retention time and accurate mass values. To determine statistically significant variations in metabolite intensities, one-way ANOVA was performed, incorporating Benjamini-Hochberg FDR correction to control for multiple testing. Identified features were subsequently matched against the METLIN metabolite database for compound annotation. UHPLC-QToF-IMS was chosen for its high resolution, sensitivity, and capability to provide accurate mass measurements along with detailed information across a wide range of metabolites.

### 2.8 Statistical analysis

Experimental data were obtained from three independent replicates and are expressed as mean ± standard deviation. Statistical analysis was performed using One-way ANOVA in Minitab software (Version 18.0). Fisher's LSD at 95 % significance level was used as post hoc test.

## 3 Results and discussion

### 3.1 Total chlorophyll content

The TCC of LJPs increased significantly under G_2_ and G_3_ conditions (Table 1). However, the increase was higher in G_3_ condition. Liu et al. (2022) conducted a study that revealed higher chlorophyll content in *Brassica* microgreens when grown under a longer photoperiod of 20 h compared to a shorter photoperiod of 12 h. The increase was approximately 19 % for *Brassica* microgreens under G_3_ condition. According to research by Hendrickson et al. (2022), the growth of herbs was significantly affected by fluctuations in root zone temperature, highlighting its importance in plant development. The study also reported that maintaining a growing temperature of 27.5 °C resulted in improved growth responses, including increased plant height, leaf area, and fresh weight. Kumar et al. (2024) demonstrated that extending the photoperiod to 22 h, combined with a temperature regime of 26 °C during the light period and 20 °C during the dark, significantly enhanced the average height and yield of *Ocimum* microgreens. Conversely, G_1_ condition caused a reduction in TCC of *Brassica* microgreens. However, the decrease was non-significance. The interaction between growth factors and the resulting yield of microgreens was strongly influenced by variations in light intensity (Jones-Baumgardt et al., 2019). Their study demonstrated that increasing light intensity (100 to 600 μmolm^−2^ s^−1^) resulted in a 65 % increase in dry weight for kale, 69 % for cabbage, 122 % for arugula, and 145 % for mustard.

**Table 1:** Effect of growing conditions on growth parameters of *Brassica* microgreens.

Growing conditions	Total chlorophyll content
Control	2.89 ± 0.15^c^
G_1_	2.81 ± 0.07^d^
G_2_	3.17 ± 0.08^b^
G_3_	3.44 ± 0.17^a^

Values with similar superscripts in a column do not differ significantly (*p* > 0.05).

### 3.2 Macroelement composition

Table 2 summarizes the macroelement composition of *Brassica* microgreens under various GCs, revealing significant variations in macroelement concentrations influenced by the different growth conditions. The concentration of sodium, potassium, calcium and magnesium increased across all conditions. In the case of *Brassica* microgreens, the macroelements (Na, K, Ca, and Mg) exhibited higher fold increase under G_3_ condition compared to G_2_ and G_1_ conditions. The content of sodium, potassium, calcium and magnesium increased approximately 2.46, 1.54, 3.03 and 1.79-fold, respectively, for microgreens. The variation in macroelement composition was attributed to the diverse mobility of nutrients under varying growth conditions, as previously elucidated. Brazaitytė et al. (2021) linked the higher proportion of blue light in the LED lighting spectrum during the development stage to the increased accumulation of primarily micro- and macronutrients in the *Brassicaceae* microgreens. The study reported Ca (14.71 mg/g), K (23.56 mg/g) and Mg (5.67 mg/g) in *Brassicaceae* microgreens which increased to Ca (16.20 mg/g), K (24.46 mg/g) and Mg (5.88 mg/g) with increase in blue light illumination. The G_1_ and G_2_ conditions led increase in the different macroelement composition, however increase was not pronounced as G_3_ condition. The plants grown in the summer season than those cultivated during the spring season demonstrated elevated mineral composition i.e., nitrogen, potassium and magnesium (Fallovo et al., 2009). The magnesium concentration was positively (moderate) correlated with TCC (*r* = 0.69). The presence of magnesium as a central component in the chlorophyll molecule may explain the positive correlation between magnesium and TCC.

**Table 2:** Effect of growing conditions on macroelement composition of *Brassica* microgreens.

Growing conditions	Macroelement composition (mg/g)			
	Na	K	Ca	Mg
Control	3.52 ± 0.12^d^	42.94 ± 0.73^d^	3.83 ± 0.64^d^	7.34 ± 0.11^d^
G_1_	4.00 ± 0.19^c^	46.24 ± 0.23^c^	5.46 ± 0.23^c^	8.54 ± 0.22^c^
G_2_	4.37 ± 0.03^b^	51.96 ± 1.56^b^	7.64 ± 0.15^b^	9.63 ± 0.29^b^
G_3_	8.69 ± 0.22^a^	66.18 ± 1.44^a^	11.62 ± 0.31^a^	13.14 ± 0.22^a^

Values with similar superscripts in a column do not differ significantly (*p* > 0.05). Na, Sodium; K, Potassium; Ca, Calcium; Mg, Magnesium. The grouping is done by the application of Fisher's LSD test (*p* < 0.05) where superscript “a” indicates the highest value among the group.

### 3.3 Phenolic profile

*Brassica* microgreens are rich in phenolic compounds, including gallic acid (GA), caffeic acid (CA), vanillic acid (VA), *p*-coumaric acid(*p*-CA), ferulic acid (FA), *t*-ferulic acid (t-FA), sinapic acid (SA), and Kaempferol (KML), in both free and bound forms (Table 3). The concentrations of these bioactive compounds varied considerably due to changes in GCs. Wu et al. (2008) found that the concentration of phytochemicals, including polyphenolics, in Chinese cabbage varied depending on the growing area, cultivar and harvest time. The major phenolic acids in Chinese cabbage leaves—caffeic, sinapic, p-coumaric, and ferulic acid—were identified via HPLC analysis (Seong et al., 2016). Under the G_1_ condition, the levels of GA and CA significantly increased. Specifically, the concentration of free GA increased by approximately 1.05-fold, while free CA concentration increased significantly (approximately 1.37-fold) under the same conditions. Phenolic compounds levels showed a positive correlation with increasing light intensity (Pennisi et al., 2020). In addition, the concentrations of free p-CA, t-FA, and KML were not significantly affected by G_1_ conditions. The study of Castronuovo et al. (2019) highlighted the impact of abiotic factors on plant characteristics, including phenotype, morphology, and yield, is profound. In *Brassica* microgreens, essential factors like shading, light intensity, and temperature regulate traits such as plant height, leaf count, length and width. Specifically, free VA and FA in *Brassica* microgreens showed a significant increase under G_1_ conditions. Significant changes in the concentration of various phenolic compounds in *Brassica* microgreens were also observed under G_2_ and G_3_. The concentration of free CA, FA and SA increased significantly under G_2_ and G_3_. The concentration of the above-mentioned compounds increased approximately 1.68, 1.87, and 1.42-fold, respectively for *Brassica* microgreens under G_2_. Likewise, G_3_ resulted in increase of approximately 2.02, 3.18, and 2.68-fold, respectively for *Brassica* microgreens. The G_3_ conditions resulted in a superior increase in the concentration of the mentioned phenolic compounds in the *Brassica* microgreens compared to G_2_ and G1. Jakovljević et al. (2021) similarly observed a rise in total phenolic, flavonoid, and anthocyanin content in *Brassica* microgreens when cultivated at 30 °C with a 16-h photoperiod, as opposed to lower temperatures. Notably, in addition to free-form phenolic compounds, bound phenolics were also present in these microgreens. Raksakantong et al. (2012) deciphered that within plants, bound phenolics were metabolic intermediates that accumulate and were retained inside membrane-bound compartments. Across all GCs, the concentration of bound CA, *p*-CA, FA, SA, and KML increased in both types of greens. Environmental stress can enhance secondary metabolite accumulation in plants, suggesting agronomic practices may boost the functional value of plant foods (Francisco et al., 2017).The microgreens exhibited the greatest increase under G_3_, while G_2_ demonstrated a moderate increase, and G_1_ showed the lowest among the three conditions. These microgreens did not contain bound chlorogenic acid and resveratrol.

**Table 3:** Effect of growing conditions on targeted phenolic profile of *Brassica* microgreens.

**Growing conditions**			**Phenolic compounds (mg/g)**								
		**GA**	**CLA**	**CA**	**VA**	***p*-CA**	**FA**	**TFA**	**SA**	**RVL**	**KML**
Control	Free	8.14 ± 0.11^d^	N·D	1.15 ± 0.04^d^	0.99 ± 0.03^d^	2.31 ± 0.12^b^	3.81 ± 0.15^d^	2.68 ± 0.14^c^	3.62 ± 0.10^d^	N·D	2.75 ± 0.16^d^
	Bound	1.38 ± 0.02^d^	N·D	0.17 ± 0.02^d^	0.06 ± 0.00^d^	0.11 ± 0.02^c^	0.97 ± 0.06^d^	0.17 ± 0.02^l^	0.47 ± 0.03^d^	N·D	0.13 ± 0.02^d^
G_1_	Free	8.66 ± 0.08^c^	N·D	1.58 ± 0.03^c^	1.57 ± 0.06^c^	2.69 ± 0.04^a^	4.90 ± 0.18^c^	3.03 ± 0.06^b^	4.00 ± 0.03^c^	N·D	3.11 ± 0.06^c^
	Bound	1.76 ± 0.01^c^	N·D	0.27 ± 0.01^c^	0.12 ± 0.03^c^	0.20 ± 0.02^b^	1.37 ± 0.01^c^	0.26 ± 0.02jk	0.70 ± 0.02^c^	N·D	0.28 ± 0.03^c^
G_2_	Free	9.40 ± 0.22^b^	N·D	1.94 ± 0.02^b^	1.91 ± 0.02^b^	N·D	7.14 ± 0.70^b^	4.24 ± 0.11^a^	5.20 ± 0.03^b^	N·D	3.79 ± 0.04^b^
	Bound	1.98 ± 0.03^b^	N·D	0.40 ± 0.01^b^	0.50 ± 0.06^b^	0.21 ± 0.03^b^	1.66 ± 0.02^b^	0.52 ± 0.01^h^	1.03 ± 0.02^b^	N·D	0.46 ± 0.01^b^
G_3_	Free	12.25 ± 0.26^a^	N·D	2.33 ± 0.12^a^	2.31 ± 0.14^a^	N·D	12.15 ± 0.91^a^	5.45 ± 0.28^a^	9.72 ± 0.11^a^	N·D	4.22 ± 0.10^a^
	Bound	2.27 ± 0.04^a^	N·D	0.73 ± 0.02^a^	0.75 ± 0.21^a^	0.35 ± 0.02^a^	2.28 ± 0.07^a^	0.96 ± 0.04^d^	1.25 ± 0.03^a^	N·D	0.68 ± 0.02^a^

Values with similar superscripts in a column do not differ significantly (*p* > 0.05). N·D, Not defined; GA, Gallic acid; CLA; Chlorogenic acid; CA, Caffeic acid; VA, Vanillic acid; *p*-CA, *p*-coumaric acid; TFA, *trans*-ferulic acid; SA, Sinapic acid; RVL, Resveratrol; KML, Kaempferol. The grouping is done by the application of Fisher's LSD test (*p* < 0.05) where superscript “a” indicates the highest value among the group. The superscripts in each column are meant to be read separately for free fractions and bound fractions.

### 3.4 Sugar profile

Table 4 summarizes the sugar profile of *Brassica* microgreens grown under different GCs. The concentrations of *myo*-inositol, glucose, fructose, and sucrose significantly increased across all GCs in *Brassica* microgreens. However, the highest fold increase was observed under the G_3_ condition, followed by G_2_ and G_1_. Specifically, under the G_3_, the concentrations of these sugars and polyalcohol increased approximately 2.02, 1.57, 2.61, and 1.78-fold, respectively. The increase in sugar content was linked to enhanced photosynthetic capacity, that may promote increased sugar synthesis under specific environmental conditions. This increase may also be attributed to the upregulation of genes involved in sugar synthesis at higher temperatures (Ruan et al., 2010). The study by Liu et al. (2020) reported changes in sugar content and metabolism during the maturation of Chinese cabbage heads. The study also deciphered the dominant presence of sucrose, glucose, and fructose. The seasonal changes in sugar content observed in our study align with the findings of Rosa et al. (2001), who reported higher levels in summer than in winter. Our findings specifically observed a significant increase in glucose concentrations in these microgreens under G_3_ during the summer season, corroborating their results.

**Table 4:** Effect of growing conditions on targeted sugar and polyalcohol profile of *Brassica* microgreens.

**Growing conditions**	**Sugars and polyalcohol (mg/g)**			
	***myo*-inositol**	**Glucose**	**Fructose**	**Sucrose**
Control	6.64 ± 0.09^d^	26.83 ± 0.44^d^	5.60 ± 0.35^d^	9.57 ± 0.31^d^
G_1_	8.15 ± 0.07^c^	34.55 ± 1.12^c^	6.02 ± 0.03^c^	10.39 ± 0.28^c^
G_2_	11.76 ± 0.23^b^	36.88 ± 0.57^b^	10.40 ± 0.20^b^	14.06 ± 0.61^b^
G_3_	13.42 ± 0.06^a^	42.27 ± 1.01^a^	14.67 ± 0.63^a^	17.08 ± 0.17^a^

Values with similar superscripts in a column do not differ significantly (*p* > 0.05).

### 3.5 UHPLC-QToF-IMS based non-targeted analysis

**Fig. 1:**
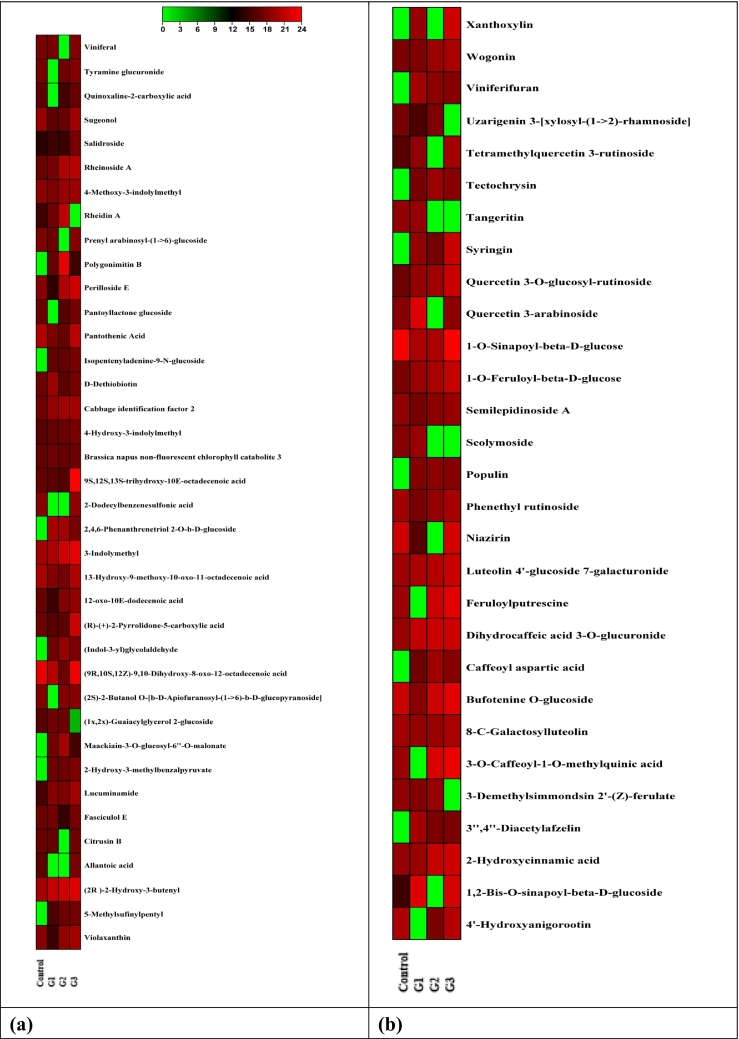

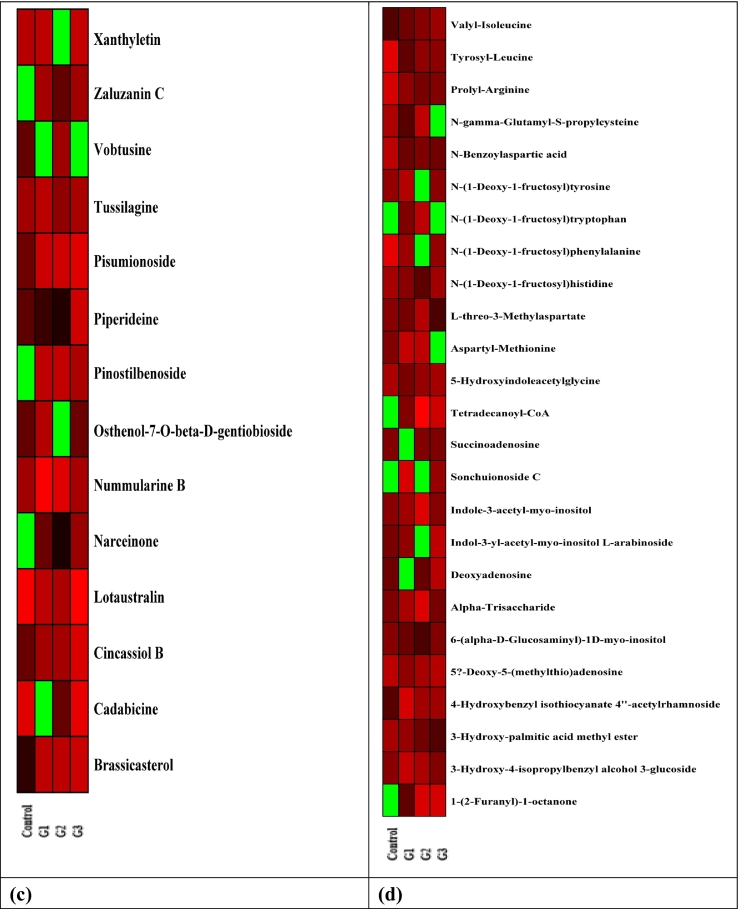
Heat map of DEMs in Chinese cabbage microgreens in different growing conditions (a.) organic acids and glucosinolates (b.) Phenolics, flavonoids and their derivatives (c.) Terpenoids, saponins, alkaloids and coumarin derivatives (d.) Amino acids, peptides, sugars, fatty acid and nucleic acid.

The comprehensive non-targeted analysis of metabolites was performed on *Brassica* microgreens grown under different GCs. The heat map of DEMs in *Brassica* microgreens under various GCs is illustrated in Fig. 1. The heat map represents metabolite concentrations, with green indicating lower levels and red indicating higher levels in increasing order. The G_3_ exhibited the highest DEMs, with 874 distinct feature peaks identified for *Brassica* microgreens. The identified metabolites encompassed different classes of compounds i.e., alkaloids, phenolic acids and their derivatives, organic compounds, terpenoids, flavonoids and their derivatives, glucosinolates, vitamins, coumarin derivatives, amino acids, nucleic acids, peptides, polyamines, sugar derivatives and fatty acids (Table 5). The pervious study by Park et al. (2020) also reported different classes of primary and secondary metabolites like organic compounds, phenolic and flavonoids, amino acids, indolic glucosinolates and aliphatic glucosinolates in leaves of Chinese cabbage herbs (*Brassica campestris* ssp. *pekinensis*). The DEMs in *Brassica* microgreens showed the presence of organic compounds like (*R*)-(+)-2-pyrrolidone-5-carboxylic acid [*m/z*–130.05], 12-oxo-10E-dodecenoic acid [*m/z*–213.15], perilloside E [*m/z*– 409.08] and violaxanthin [*m/z*–601.97]. The volume of (*R*)-(+)-2-pyrrolidone-5-carboxylic acid increased significantly under G_3_. However, G_1_ and G_2_ showed downregulation of the same compound. Again, violaxanthin, a product of epoxidation of zeaxanthin also showed upregulation in the volume under G_2_ and G_3_. However, more pronounced increase was noted under G_3_ as compared to G_2_. Pfündel and Bilger (1994) also reported that the violaxanthin cycle adapted to varying environmental conditions over both short and extended periods, regulating the rates of pigment conversions and the pigment pool sizes. Park et al. (2020) also reported the presence of violaxanthin in in leaves of Chinese cabbage herbs. Furthermore, the expression level of 12-oxo-10E-dodecenoic acid and perilloside E also showed upregulation in G_2_ and G_3_, whereas G_1_ led downregulation of the mentioned compounds. In addition, Wang et al. (2022) reported the antioxidant activity of 12-oxo-10E-dodecenoic acid. Another class of compounds in *Brassica* microgreens included the presence of glucosinolates. Glucosinolates represent a vast collection of plant secondary metabolites containing both nutritional impacts and biologically active elements. These compounds were predominantly located within cruciferous plants, notably in the *Brassicaceae* family, encompassing widely consumed vegetables like cabbage (Prieto et al., 2019). The expression level of detected glucosinolates i.e., (2*R*)-2-hydroxy-3-butenyl [*m/z*– 390.52], 4-hydroxy-3-indolylmethyl [*m/z*–464.52] and 3-indolymethyl [*m/z*–132.28] upregulated across all GCs. However, more prominent upregulation was recorded under G_3_ followed by G_2_ and G_1_. Another study by Liu et al. (2014) reported the presence of progoitrin [(2*R*)-2-hydroxy-3-butenyl], 4-hydroxyglucobrassicin [4-hydroxy-3-indolylmethyl] in different varieties of Chinese cabbage. The increase or decrease in specific metabolite levels relied on the characteristics of the compound exposed to the corresponding GCs. Park et al. (2019) highlighted that the impact of varying light sources and wavelengths on the synthesis of natural compounds could rely on factors such as the type of plants and cellular composition. Their research held the prospect that the implementation of LED lighting could augment the yield of glucosinolates in *Brassica napus*. Another study by Jasper et al. (2020) concluded that higher growing temperature led more accumulation of glucosinolates in rocket salad. Furthermore, phenolic acids, their derivatives, and flavonoids were identified in *Brassica* microgreens cultivated under different conditions. The expression levels of these metabolites varied significantly in response to changes in the GCs. The primary phenolic derivatives detected included 2-hydroxycinnamic acid [*m/z*–182.08] and 1-O-feruloyl-β-d-glucose [*m/z*–379.10]. The aforementioned compounds showed upregulation in the expression level across all conditions. However, the higher increase was observed under G_3_ followed by G_2_ and G_1_. The metabolomic profile of LJP of Chinese cabbage herb consisted of various metabolites, among which phenolic acids were identified, as reported by Park et al. (2020). Furthermore, the study established the existence of gallic acid, caffeic acid, trans-cinnamic acid within the group of phenolic acids. Also, a coumarin derivative i.e., xanthyletin [*m/z*–229.09] showed increased expression level under G_1_ and G_3_. However, higher fold increase was recorded under G_3_ and it was not detected in G_2_. In a study, Li et al. (2018) documented that the primary categories of phenolic compounds present in cruciferous vegetables consist of hydroxycinnamic acids such as ferulic, caffeic, *p*-coumaric, and sinapic acids, were identified. These acids were observed to be conjugated with sugars or other hydroxycinnamic acids, serving both structural and chemical roles in the plant's defense strategies. The flavonoids are identified for their free-radical scavenging capacity, antioxidative activity, anticancer activity and coronary heart disease prevention (Yao et al., 2004). The prominent flavonoid derivatives screened in *Brassica* microgreens were luteolin 4′-glucoside 7-galacturonide [*m/z*–647.12], quercetin 3-O-glucosyl-rutinoside [*m/z*–795.19] and wogonin [*m/z*–285.07]. The volume of aforementioned metabolites also increased across all GCs. However, higher fold increase in the volume of mentioned metabolite was recorded under G_3_ followed by G_2_ and G_1_. In a study by Sun et al. (2013), a total of 165 phenolic compounds were identified within *Brassica* herbs. This identification was achieved through the integration of complementary data obtained from UHPLC-PDA-HRMS^n^ techniques. The results of the study unveiled a substantial abundance of highly glycosylated and acylated forms of quercetin, kaempferol, cyanidin aglycones, as well as intricate hydroxycinnamic and benzoic acids. Notably, the research also highlighted that *Brassica* species exhibited more intricate polyphenol profiles and encompassed a greater diversity of polyphenol types. The concentration of scolymoside [*m/z*-579.16], a flavonoid derivative, rose under G_1_, while it dropped under G_2_ and G_3_. Seong et al. (2016) observed that extended exposure to light resulted in a rise in phenolic compounds (*p*-coumaric, ferulic, and sinapic acids) and the flavonoid myricetin within leaves of Chinese cabbage. Light perception initiates a series of molecular signaling events that regulate the expression of circadian clock-associated genes in a time-specific manner throughout the day (Pokhilko et al., 2012). Jan et al. (2021) deciphered that the bHLH transcription factors were potential regulators of stress response mechanisms and usually interact with proteins to generate complexes that enhance the expression of specific genes. The modulation of phenolic and flavonoid derivatives in response to varying GCs served as a defensive response triggered by stress in plants. Moreover, photoperiod and growing temperature, a widely recognized instigator of physical stress, directly influenced physiological processes and the synthesis of secondary metabolites across developmental stages. This influence resulted in the augmentation or reduction of metabolite levels. Managa et al. (2020) conveyed that heightened synthesis of kaempferol in Chinese cabbage could be attributed to summer temperatures and intensified light radiation, alongside glycosylation with sugar molecules, sophorotriose formation, and ferulic acid-driven acylation. Høyen (2017) also reported that variations in photoperiod and temperature influenced the metabolite composition of several microgreens—including beet greens, peppercress, lettuce, and wheatgrass—thereby impacting both their flavor profiles and associated health benefits. *Brassica* microgreens have been reported to contain high levels of bioactive compounds, with their concentration being significantly affected by environmental factors and cultivation practices (Allogia et al., 2025). The metabolomic profile of *Brassica* microgreens were diverse, featuring alkaloids and terpenoids. These metabolites displayed significant expression changes in response to GCs, with piperideine [*m/z*–84.08] upregulated in G_3_. However, G_1_ and G_2_ caused a reduction in expression level. Liu et al. (2014) identified piperideine in *Brassica* species and highlighted that secondary metabolic pathway, including the biosynthesis of indole alkaloids and tropane, pyridine and piperidine alkaloids, originate as a defense stress response. Mitra et al. (2022) further underscored piperidine's potential as a therapeutic agent for cancers such as prostate, breast, colon, ovarian, and lung. Additionally, *Brassica* microgreens were found to contain significant metabolites such as pisumionoside [*m/z*–427.20], identified as terpenoids. The levels of these terpenoid derivatives were upregulated under the growing conditions G_1_, G_2_, and G_3_, with the highest increase observed under G_3_, followed by G_2_ and G_1_. Similarly, Arena et al. (2020) identified various classes of phytochemicals in *Brassica juncea* using RP-LC-PDA-MS. Progressing further, the phytochemical analysis of *Brassica* microgreens also marked the presence of peptides and amino acids derivatives like cabbage identification factor 2 [*m/z*–329.07], tyrosyl-leucine [*m/z*–295.16] and N-benzoylaspartic acid [*m/z*– 294.15]. The volume of aforesaid peptide and amino acid derivatives showed downregulation across all GCs. On the contrary, prolyl-arginine [*m/z* – 294.15] exhibited increased expression across all GCs. In a study, Gmižić and Šola (2025) deciphered that the high-temperature stress significantly affected mature broccoli heads, with proline levels exhibiting the most pronounced response—showing an increase of up to 168 %. Park et al. (2020) also elucidated that several amino acids i.e., phenylalanine, tryptophan, and methionine in Chinese cabbage herbs.

**Table 5:** Effect of growing conditions on non-targeted metabolomic profile of *Brassica* microgreens.

**t**_**R**_**(min)**	**Molecular Formula**	**Measured Mass**	***m/z***	**Proposed Identification**	**G**_**1**_	**G**_**2**_	**G**_**3**_	**Class**	**Bioactive functions**	**Reference**
1.6	C_15_H_18_O_10_	358.09	381.08	Dihydrocaffeic acid 3-O-glucuronide	+	+ +	+ + +	Phenolic glucoside	Improve glucose metabolism	Gao et al. (2023)
1.7	C_5_H_7_NO_3_	129.04	130.05	(*R*)-(+)-2-Pyrrolidone-5-carboxylic acid	−	−	+	Organic acid	−	−
1.8	C_5_H_9_N	83.07	84.08	Piperideine	−	_−_	+	Alkaloid	Anticancer activity	Mitra et al. (2022)
1.9	C_11_H_19_NO_6_	261.12	262.13	Lotaustralin	+ +	+	+ + +	Cyanogenic glucoside	Antioxidant activity	Schmidt et al. (2018)
2.0	C_10_H_13_N_5_O_3_	251.10	274.09	Deoxyadenosine	_+_	−	N·D	Nucleoside	−	−
2.4	C_9_H_8_O_3_	164.05	182.08	2-Hydroxycinnamic acid	+	+ +	+ + +	Phenolic acid	Anti-inflammatory activity	Taofiq et al. (2017)
2.9	C_9_H_17_NO_5_	219.11	220.12	Pantothenic Acid	+	−	+ +	Vitamin	CoA synthesis	Khan and Khan (2022)
3.8	C_40_H_56_O_4_	600.92	601.97	Violaxanthin	−	+	+ +	Pigment	Radical scavenging activity	Wang et al. (2018)
4.6	C_33_H_40_O_21_	772.21	795.19	Quercetin 3-O-glucosyl-rutinoside	+	+ +	+ + +	Flavonoid glycoside	Antioxidant activity	Pyo et al. (2021)
5.9	C_28_H_46_O	398.69	399.7	Brassicasterol	−	^+^	+ +	Sterol	Anti-infective property	Hassan (2020)
6.9	C_16_H_20_O_9_	356.11	379.10	1-O-Feruloyl-β-d-glucose	+	+ +	+ + +	Phenolic acid derivative	−	−
7.1	C_15_H_12_N_4_O_3_S	328.06	329.07	Cabbage identification factor 2	−	−	−	Peptide	−	−
7.4	C_17_H_22_O_10_	386.12	409.11	1-O-Sinapoyl-β-d-glucose	+	+ +	+ + +	Phenolic acid derivative	−	−
8.3	C_16_H_19_NO_7_	337.13	360.10	Indole-3-acetyl-*myo*-inositol	+	+ +	+ + +	Sugar derivative	−	−
9.2	C_11_H_19_NO_10_S_2_	389.45	390.52	(2*R*)-2-Hydroxy-3-butenyl	+	+ +	+ + +	Glucosinolate	−	−
12.5	C_12_H_20_O_3_	212.14	213.15	12-oxo-10E-dodecenoic acid	N·D		+	Organic acid	Promotes SOD activities	Wang et al. (2022)
15.5	C_17_H_34_O_3_	286.25	309.24	3-Hydroxy-palmitic acid methyl ester	+	−	−	Fatty acid derivative	−	−
15.7	C_16_H_19_N_2_O_10_S_2_	463.51	464.52	4-Hydroxy-3-indolylmethyl	+	+ +	+ + +	Glucosinolate	Chemoprotective effect	Maina et al. (2020)
16.2	C_12_H_20_O_8_	292.12	293.12	Pantoyllactone glucoside	+	−	N·D	Fatty acyl glycoside	−	−
17.2	C_9_H_9_N	131.20	132.28	3-Indolymethyl	+	+ +	+ + +	Glucosinolate	Antimicrobial activity	Zeng et al. (2003)
18.9	C_14_H_12_O_3_	228.08	229.09	Xanthyletin	+	N·D	+ +	Coumarin derivative	Antifungal activity	Wittayapipath et al. (2019)
19.6	C_17_H_22_N_2_O_10_S_2_	478.52	479.58	4-Methoxy-3-indolylmethyl	+	+ +	+ + +	Glucosinolate	Antioxidant activity	Chuanphongpanich et al. (2006)

t_R,_ Retention Time; *m/z*, mass-to-charge ratio; +, upregulation; −, downregulation. The “+++” indicates many folds upregulation of the compound; N·D, Not defined.

In *Brassica* microgreens, metabolomic analysis further confirmed the presence of sugar derivatives, including 1-O-feruloyl-β-d-glucose (*m/z* – 379.10) and 1-O-sinapoyl-β-d-glucose (*m/z -* 409.11). These sugar derivatives exhibited increased expression across all GCs, with the highest levels observed in G_3_, followed by G_2_ and G_1_ conditions. The analysis also revealed the presence of fatty acids and nucleic acid derivatives.

## 4 Conclusion

The study investigated the impact of varying GCs on the metabolomic profiles and overall nutritional quality of *Brassica* microgreens. The findings demonstrated that a prolonged photoperiod of 22 h combined with specific temperature settings (26 °C/20 °C:: Light/Dark) substantially enhances chlorophyll content, microgreen height, and macroelement composition, particularly increasing potassium levels. The targeted analysis revealed that G_3_ prominently elevate key phenolic compounds like gallic and ferulic acid, as well as sugars such as glucose. The non-targeted metabolomic profiling further underscored the upregulation of various beneficial compounds under G_3_ condition. These insights pave the way for optimized agricultural practices that can boost the nutritional and functional qualities of *Brassica* microgreens, offering promising implications for both growers and consumers aiming for enhanced dietary benefits. Also, there is need to validate bioactivity of identified metabolites. Additionally, integration of omics-based selection with precision agriculture may further advance the development of resilient and nutrient-dense microgreen varieties tailored to diverse environments.

## CRediT authorship contribution statement

**Arun Kumar:** Writing – review & editing, Writing – original draft, Resources, Formal analysis. **Narpinder Singh:** Writing – review & editing, Software, Project administration, Funding acquisition. **Robin Joshi:** Software.

## Ethical approval

Ethics approval was not required for this study. The experiments were not conducted on humans or animals.

## Declaration of competing interest

The authors declare that they have no known competing financial interests or personal relationships that could have appeared to influence the work reported in this paper.

## Data Availability

Data will be made available on request.

## Appendix A Supplementary data

Supplementary material: Corresponding growing conditions for the mentioned treatments. Image 1
