# Supplementary material for: Enhancing Brassica microgreen production: Exploring metabolomic variations across growing conditions using targeted and non-targeted analysis
Source: Food Chem X. 2025 Oct 10;31:103125. doi: 10.1016/j.fochx.2025.103125 (PMC12552544; doi:10.1016/j.fochx.2025.103125)
Supplement: Supplementary file 1 — Supplementary material: Corresponding growing conditions for the mentioned treatments. [file mmc1.docx]

**Appendix: A**

**Supplementary data**

**Table S1:** Corresponding growing conditions for the mentioned treatments.

| **Treatments** | **Growing Conditions (Photoperiod and temperature combination)** |
| --- | --- |
| Control | Photoperiod (16 h Light: 8 h Dark) & Temperature (22 ^o^C in light and 17 ^o^C in dark) |
| G_1_ | Photoperiod (22 h Light: 2 h Dark) & Temperature (22 ^o^C in light and 17 ^o^C in dark) |
| G_2_ | Photoperiod (16 h Light: 8 h Dark) & Temperature (26 ^o^C in light and 20 ^o^C in dark) |
| G_3_ | Photoperiod (22 h Light: 2 h Dark) & Temperature (26 ^o^C in light and 20 ^o^C in dark) |
